# Supplementary material for: The Intersection of Human Disturbance and Diel Activity, with Potential Consequences on Trophic Interactions
Source: PLoS One. 2019 Dec 13;14(12):e0226418. doi: 10.1371/journal.pone.0226418 (PMC6910683; doi:10.1371/journal.pone.0226418)
Supplement: S1 Table — (PDF) [file pone.0226418.s001.pdf]

S1 Table. Specific locations of camera traps in coastal Orange County, California, 2007–2016.

Camera	Latitude	Longitude
AG CH	33.70846	-117.67305
AU TR	33.75832	-117.70193
BG_BR	33.60528	-117.84944
BG_GC	33.60344	-117.83627
BG_PCN	33.59948	-117.84636
BG_PPD	33.58766	-117.85524
BG_SJH1	33.61098	-117.84168
BG_WE	33.60451	-117.8388
BO SP	33.7223	-117.70988
BO TR	33.71725	-117.67283
CA CR	33.6275	-117.78167
CO MI	33.80553	-117.7098
CO TR	33.7606	-117.71812
DO CA	33.82803	-117.67673
DO CA3	33.82778	-117.6775
DR SP	33.73408	-117.68108
DR SP2	33.73369	-117.68092
EA MW2	33.79472	-117.72917
FR RO	33.79029	-117.72311
FU BR	33.75892	-117.70265
FU TR(BC)	33.61462	-117.80197
GY FO	33.85083	-117.70494
LA RO	33.79433	-117.68388
LI ME2	33.7425	117.67833
LI SI	33.71722	-117.67278
LO EA	33.60694	-117.79278
LO WE2	33.8192	-117.74698
ME SP	33.72018	-117.66135
MI LI	33.63477	-117.77322
MO FR1	33.792	-117.72055
MO FR3	33.79163	-117.71995
MU DE	33.62928	-117.76643
OR LO	33.755	-117.74178
OV TR	33.82955	-117.72083
RA TR	33.739	117.67372
RO CA2	33.70944	117.69444
SE RI	33.62037	-117.76752
SO GY	33.83828	-117.71252
TH SI2	33.61444	-117.78056
TUR PND	33.60693	-117.77972
UP WE	33.83475	-117.73025
WE FO	33.6124	-117.80047
WE FO2	33.61336	-117.8006
WE SP	33.82678	-117.74347
WE TR1	33.8246	-117.74387
WE TR2	33.82988	-117.74082
WE TR3	33.83303	-117.73563
WE WI	33.8336	-117.68335
