# Supplementary material for: The Intersection of Human Disturbance and Diel Activity, with Potential Consequences on Trophic Interactions
Source: PLoS One. 2019 Dec 13;14(12):e0226418. doi: 10.1371/journal.pone.0226418 (PMC6910683; doi:10.1371/journal.pone.0226418)
Supplement: S1 Fig — (PDF) [file pone.0226418.s002.pdf]

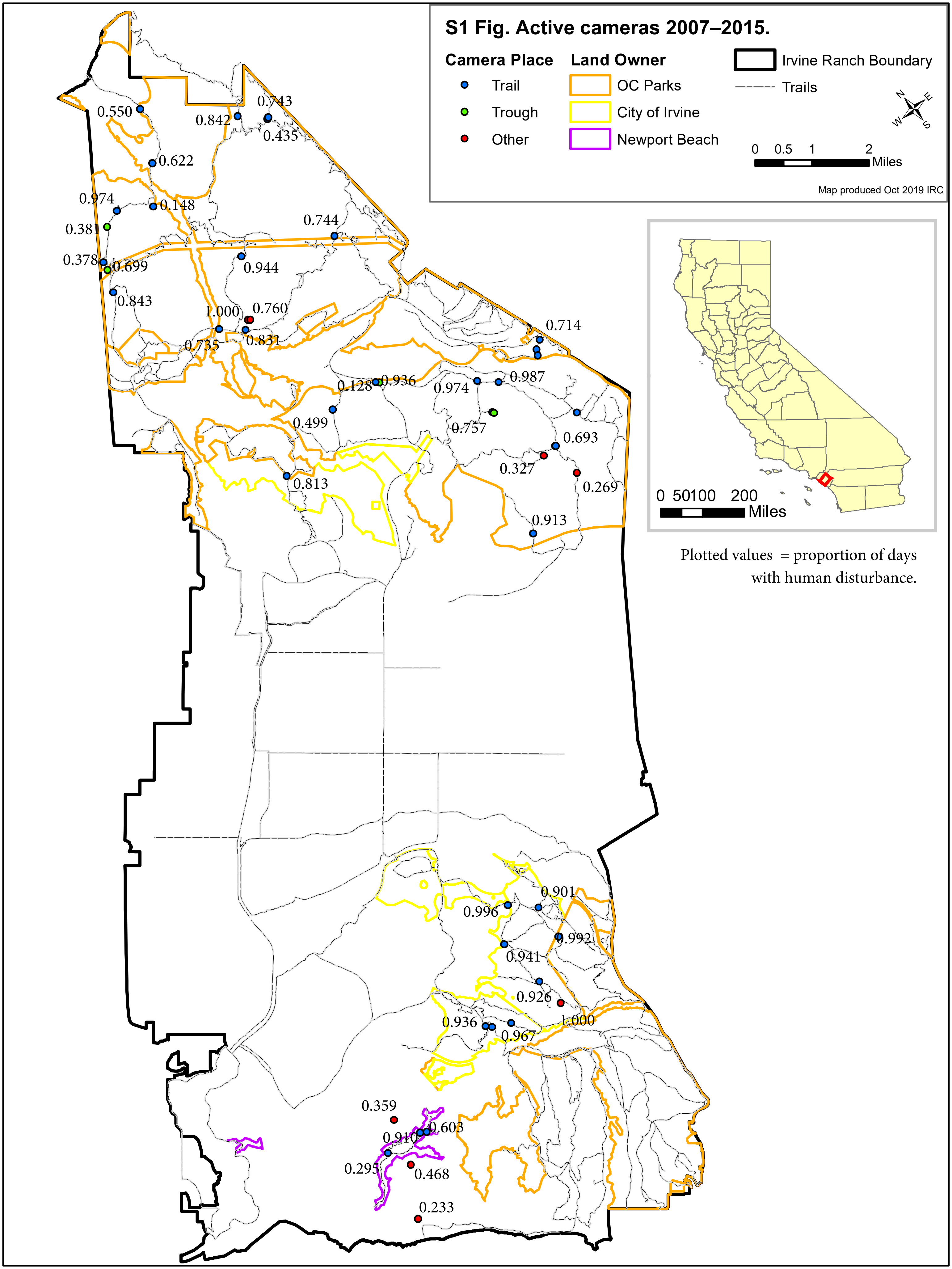

S1 Fig. Active cameras 2007–2015.
