# Supplementary material for: The Intersection of Human Disturbance and Diel Activity, with Potential Consequences on Trophic Interactions
Source: PLoS One. 2019 Dec 13;14(12):e0226418. doi: 10.1371/journal.pone.0226418 (PMC6910683; doi:10.1371/journal.pone.0226418)

S2 Fig. Parameter estimates of peak activity of the Mule Deer (*Odocoileus hemionus*) and Coyote (*Canis latrans*) when camera trap images are excluded within 1 hour of the previous image of that same species or only within 5 minutes of the previous image. Estimates are of mean activity +/- credible intervals from a Bayesian model in which data likelihood followed a von Mises distribution.

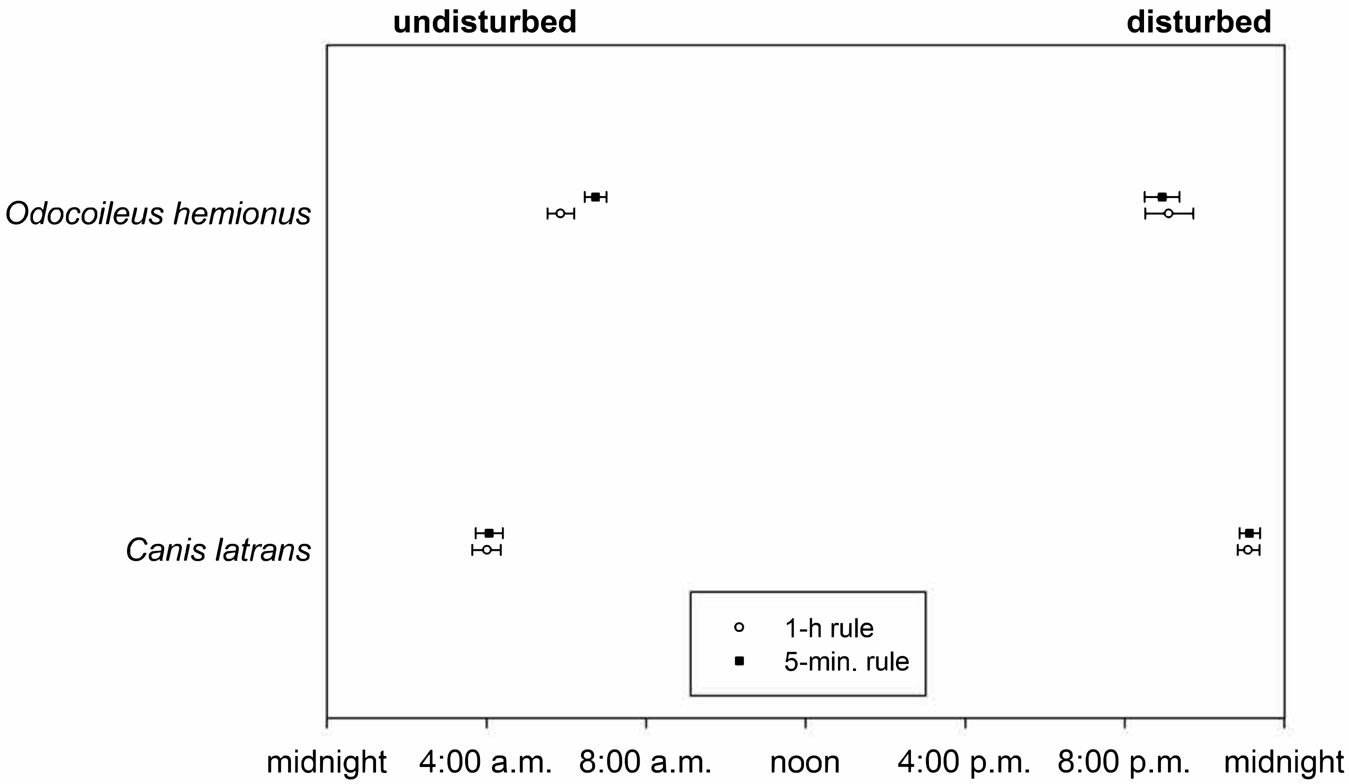

Supplement: S2 Fig — (PDF) [file pone.0226418.s003.pdf]
