# Supplementary material for: The Intersection of Human Disturbance and Diel Activity, with Potential Consequences on Trophic Interactions
Source: PLoS One. 2019 Dec 13;14(12):e0226418. doi: 10.1371/journal.pone.0226418 (PMC6910683; doi:10.1371/journal.pone.0226418)

S3 Fig. Temporal distributions for human activity in this study. Note the distinct peak in mid to late morning as well as the near absence of activity after dark.

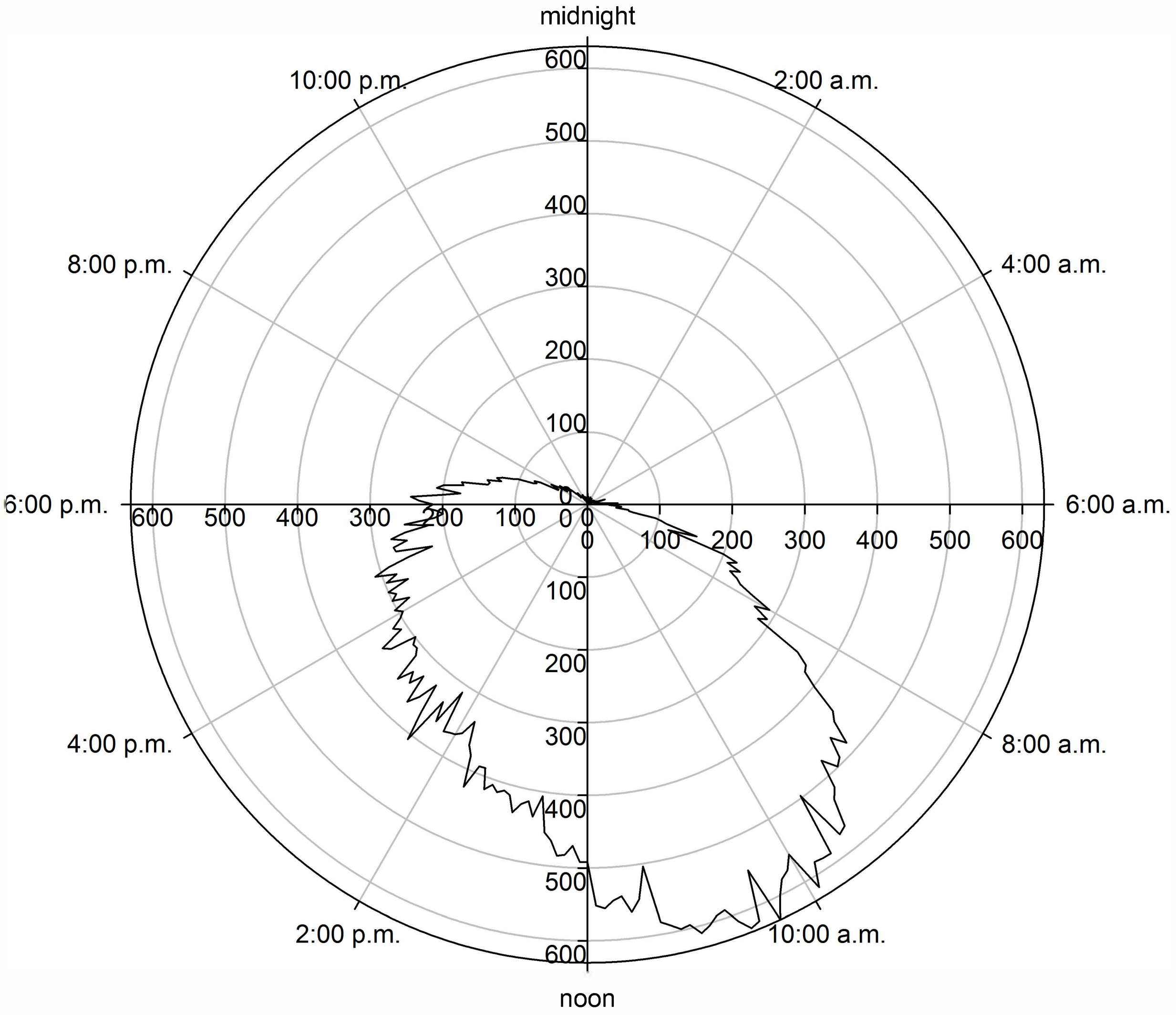

Supplement: S3 Fig — (PDF) [file pone.0226418.s004.pdf]
