# Supplementary material for: The Intersection of Human Disturbance and Diel Activity, with Potential Consequences on Trophic Interactions
Source: PLoS One. 2019 Dec 13;14(12):e0226418. doi: 10.1371/journal.pone.0226418 (PMC6910683; doi:10.1371/journal.pone.0226418)
Supplement: Attachment — Submitted filename: letter1.pdf [file pone.0226418.s006.pdf]

Oklahoma Biological Survey
111 E. Chesapeake Street
University of Oklahoma
Norman, Oklahoma 73019
Lara Souza, Director

Telephone (405) 325-5061
FAX (405) 325-7702
E-MAIL mpatten@ou.edu

24 August 2019

Paulo Corti, Ph.D.
Academic Editor, *PLoS ONE*

Dear Dr. Corti,

My coauthors and I have revised our manuscript titled “The intersection of human disturbance and diel activity, with potential consequences on trophic interactions,” which we had submitted to *PLoS ONE* for potential publication. Herein we detail how we responded to referee comments and critiques.

Reviewer #1

The authors analyze a very large data set of large-mammal pictures taken by authomatic cameras in protected areas in California. The authors look at temporal patterns of activity to analyze if human presence is coupled with a shift in anuimal activity, and if this kind of changes can change predator-prey and agonisitic behaviour/intra-guild predation risks. Data are robust and methods in general appropriate, though some improvements are needed. See more detailed comments below.

- Please, include in results the diel activity graph of human activity. It is central to your discussion and readers only know that human activity is 95% diurnal. Also, indicate the percentage of disturbed vs. Undisturbed days of your cameras, and the variability among them.
Response: I added the polar graph we had created to depict human activity. It was not clear to me if the reviewer wished to see the proportion of disturbance at each camera or if a statement of average disturbance and associated variability would suffice. We opted for the latter, which now is reported at the beginning of the Results.

- The methodological observation in l. 120-125. pinpoints something rather relevant that has to be taken into account, discussed and potentially analyzed in the paper. The consistency of results

either defining the 'disturbed/undisturbed' as presence of human activity before or after the animal detection shows that it is not the case that the (recent) presence of human disturbance shapes the response you describe in the paper. Thus, the differences in diel patterns you find must derive from a correlation/response at a higher level, either temporal or spatial.
In the case that there is 'temporal correlation in your observations' (the same camera takes repeatedly pictures from the same animal species with different beahaviours -disturbed/undisturbed-, but observation of the former and latter are aggregated), your results show that the behaviour of animals changes seasonally in sites visited by humans during one season (e.g. Easter vacation, summer…). That's why you find that it is the same to analyse data looking to the previous or the next day (most probably both are high- or low-human occupancy)
The other option (spatial correlation) is that you always get animals in 'disturbed' conditions in some points (e.g. cameras located closest to parking lots at entrances) and 'undisturbed' animals always in other sites (deeper in the reserve where fewer hikers reach). In this case, you may have individuals that always behave in one of the two conditions.
<u>Response</u>: I read and re-read this critique in an effort to wrap my head around it. It occurred to me only after several reads that, presumably from our ambiguous wording, the reviewer misconstrued our methodology. At no point did we use human presence *after* mammal detection as a means to categorize disturbance (i.e., there was no "presence of human activity before or after the animal detection"—human activity was always before and never after). I added some wording to this section to clarify this point, and I created a rough map (for SOM) that displays the proportion of days with human presence across most of the cameras (I did not have a base map with all of the cameras, so a few are omitted, but the general pattern would not change).

The above does not invalidate your research at all, but it is rather important to know about it to understand the potential implications. I suggest analysing the spatial and temporal distribution of disturbance as well as those of animal observations, and including descriptive data on that in the paper to properly discuss the issue. In a (somewhat) similar vein, I suggest informing also the frequency of cameras with detections of species in pairs for a somewhat narrower time window (e.g. number of coincidences in season and site of puma-Odocoileus). All this information can be very valuable and worth discussing.
<u>Response</u>: Because of the misunderstanding, albeit preventable with clearer wording, I expect this comment is now moot and, thus, a separate spatial analysis, which would greatly complicate the paper and its message, is unnecessary. I did, however, calculate expected vs. observed occurrences by camera of deer vs. Puma and added those data in a separate table.

- l. 125-129. I suggest revising the use you do (in results and discussion) to the concept of 'peak activity' and 'change in peak activity' due to the statistics involved. The Rayleigh's test formally gives you the presence of directionality in the clock vs. randomness, but it is not fully informative of 'a peak hour' (see Odocoileus case in Figure 2a for a clear case of 'peak from a bi-modal distribution'). The point with this test is that it computes an average vector for all

observations and analyses if its length is larger than expected under random conditions (some bi-modalities may even show lack of peak!!). The second analysis based on ranks (it is not fully explained but from my experience with circular statstics I guess the details) also looks for differences in 'temporal distribution' rather than differences in 'peak'. As a conclusion, I suggest better dealing with it as changes in temporal distributions.
Response: For the cogent reasons the reviewer noted, I removed all reference to "peak activity" and refer instead to "temporal distribution" or some variant thereof.

- l. 148-150. Do not include the option for 'spatial avoidance' since you can't say about that with this approach: In case prey spatially avoided predators, then their presence in other cameras would compensate for this movement. The case that they 'move away' of your cameras (but they stay in the area) can't be discussed on scientific grounds (you would need a posteriori explanations based on imperfect sampling with your cameras added to a biased presence of predators close to your cameras). Moreover, with your data you can't blame changes in diel activity as responsible in population abundances. You just describe a reduction in deer numbers occurring at the same time that puma numbers increase, but we do not have any clue about the reason (ok, it can be increased predation, but higher numbers of puma simply predate more to survive and we are not informed if diel activity of deer has gradually changed since 2007 until 2016, or about changes in attack success of puma along the years)
Response: I do not disagree with the reasoning in the first sentence, but I would counter that this reasoning holds only if cameras are positioned randomly or systematically across the landscape. Alas, neither was the case here. I nonetheless removed the offensive clause and reworked the sentence to avoid any attribution to potential cause.

- Methods in l. 143-153need to be clarified (and/or re-organized). I understand (right? wrong? And readers should not face many doubts) that you first did a kind of Bayesian occupancy modelling to estimate probability of detection and presence using raw 0-1 data. From that you extract the slopes –interannual change- (those painted in fig. 3? Why don't you include a second y-axis scaled accordingly?) and their plausabilities of being different from 0. After you have the detection probabilities for each year, you use the raw data of number of detections per quarter per camera corrected for detectability to compute abundance data. Finally, it is not clear if lines in fig 3 are just regression lines fitted to the (log-)abundance data and drawn ignoring any kind of their fit statistics. In short: explain it a bit better and in the logical order of construction (I may have passed several issues).
Response: An *N*-mixture model is, in effect, an occupancy model in which the response variable is abundance rather than presence/absence. I explained this in more detail, in that I made an effort to step back to be a bit more didactic about the analysis. I explored the idea to add a second *y*-axis label but ultimately failed to hit upon a solution that I felt was satisfactory, chiefly because all of the data in the figure are on the same (albeit log) scale, and, as noted in the figure caption, the lines are mere "decoration," there to help a reader see the pattern. The results spelled out in

the caption are from a beta regression on occupancy probability.

- I encourange including (it can be as supplementary) the diel activity graphs for all species in both situations (one figure by species).
Response: I added SOM graphs for the three other species not included in Fig. 1 or Fig. 2.

Other comments

- Include a map of the study area with the distribution of protected areas-working sites and urban areas (not needed in a very precise detail that may compromise the robbery of cameras)
Response: There is now a map in SOM. We noted in the Methods that a map of camera locations and the study area appeared in Patten and Burger (2018), and it still strikes me that we need not reproduce the same map in print.

- l. 108-111. Further justify why you do not use the 1-h lapse time between animal snaps to differentiate ‘animal visits’ since it is the time window usually applied in studies of carnivores. Moreover, two observations of the same species in the same place in 5-10’ when you work with large mammals (puma, deer) will correspond in most cases to the same individual just moving around (e.g. puma) or feeding (deer). On the contrary, you applied the one-hour rule for people. It would be also needed to know how many observations you have that do not fit the one-hour rule but only the 5’ one.
Response: I calculated that ~15% of mammal records would not meet the “1-h rule”; I added this information to the text.

- Figures 1 and 2 apparently show data by 30’ intervals. If this is the case (in order to somewhat smooth their appearance) explain it. In the caption, substitute ‘…relative to human disturbance’ by something like ‘… under the disturbance of human and its absence’. The present captions give the idea of confronting in the same plate diel activity of the animals and human activity.
Response: I changed wording to “with or without human disturbance” and added a statement about binning the data at 30-min. intervals.

- You can easily compute the percentage of overlap shown in figures 1 and 2, and include in in results. This features will have the advantage of being rather easy to understand for readers.
Response: I do not feel it is easy to calculate overlap, apart from program ‘overlap’ in R, which is designed to do just that. I have concerns about this package, though, because it does not account for the circular nature of the data, and hence I have not reported results from it. I still do not feel this paper is the place to air concerns about that approach, particularly because Fig. 1 and Fig. 2 “speak for themselves,” as the old trope goes.

Typographical errors and other minor issues

- l. 137-139. In fact you compute 'the odds of two odd ratios'. I do not know if it could be better to find another name or explain it better (it is a suggestion, it first surprised me and I took a bit time to understand it… but I am not English native)
Response: I do not know that there is a better way, so I adopted the suggested wording.

- l. 192 … potential increase in encounter rate
Response: I may not have understood this comment, but I adjusted wording to say "a predator's potential functional response. . . ."

- Table 1. Do not include second in the peak time. Use the same number of decinal positions in all 'P' values
Response: done

Reviewer #2

Patten et al. use a long-term camera trapping dataset to evaluate shifts in species diel activities in response to human disturbance and assess the consequences for predator-prey relationships. The question being addressed – the influence of humans on ecological interactions – is important, and the data used are extensive. The manuscript is clearly written overall.

However, I have three major concerns with this manuscript:

1) A prey species can share the same diel pattern that its predator, yet avoid the latter in space. This mechanism is hinted at in the Puma-Mule Deer analysis, but I am not convinced by that analysis (see point 2 below). Until the authors show that these diel shifts are or aren't accompanied by changes in space use, conclusions regarding changes in predation risk are unfortunately not supported. Unless I have missed something, I do not see this kind of analysis in the manuscript.
Response: I regret that we cannot address this comment. Our data set is not such that we may demonstrate the presence or absence of a spatial shift. (Reviewer #1's response to this issue was to suggest that we remove reference to a spatial shift. I did remove it, even if I worry now that it may appear that we did not consider the matter.) If I have not misinterpreted, the reviewer thinks it possible that the disturbance-mediated temporal shift we think we see is an illusion wrought by a disturbance-mediated spatial shift away from detection. I do not think that alternative is plausible because we so no evidence for a change in detection probability of the species (noted in the caption for Fig. 3) but instead see an apparent change in abundance.

2) Using the "peak activity" as the sole metric with which to compare activity distributions seems a bit restrictive. What if the distribution is bimodal, hypothetically?

Response: I changed wording from “peak activity” to “temporal distribution” and in doing so now emphasize that we compare means, a tried-and-true if admittedly restrictive, approach. From the outset I attempted to be transparent about the shifts in the distributions themselves via the way I constructed Fig. 1 and Fig. 2, which I feel visually emphasizes the shift not just in the mean occurrence (formerly “peak activity”) but shows the spread in the data, including the modality.

3) The analysis of abundance time series could be improved. First, given the cyclic and non-independent nature of the time series, I’m not sure a simple linear regression is the most appropriate approach to quantify trends. On top of this, there is added variation due to seasonality/quarter that does not seem to be accounted for. Second, why not implement a single model assessing prey abundance as a function of predator abundance, human disturbance, with random effects to account for year and quarter. The temporal unit would be the quarter. Another approach could be to look at cross-correlations (possibly lagged) between the time series considered. Overall, I admit that this analysis was a bit unclear to me.
Response: I have improved the explanation of the methodological approach. At some point I ran a time series analyses of these data, binned by quarter and with cross-correlational plots. I do not report those results herein because, well, time-series analysis is notoriously difficult to explain clearly and well, and most readers do not find cross-correlational plots at all easy to parse. In the end, though, that analysis did not alter the findings as I present them here. Instead, I feel that the method I employed, and that I hope I have explained better, is easier for a reader to grasp. (I say this from experience presenting these results to stakeholders in various local, state, and federal agencies and NGOs. The visual aids and more straightforward analyses were effective; the time-series analyses and associated non-linear regressions elicited little more than blank stares.)

Minor comments:
l. 28 – not clear what “avoidance can mirror predation” means here. Could you please clarify or reword?
Response: I rewrote to say that “avoidance can have the same ecological effects as predation.”

l. 52 – “basic ecological terms” is a bit vague. What would these be?
Response: I deleted this clause; in re-reading it, it strikes me as superfluous given the opening sentence of the next paragraph.

l. 92 – Cuddeback instead of “Cuddleback”
Response: corrected

l. 108-11 – I think the difference in independence thresholds needs justifying.
Response: I added more information about this in response to reviewer #1.

l. 117-9 - why wasn’t the disturbance caused by the researchers setting/checking the cameras also

included?
Response: Data were downloaded only when activity, both human and large(r) mammals, was at a minimum and never took longer than a few minutes per camera. Moreover, for the bulk of the study data were gathered only quarterly, with maintenance done when cameras were inoperative.

l. 143 – a reference to support this statement is needed
Response: A reference is provided already in the Introduction [31].

l. 164-66 – use of the present tense is awkward, I suggest changing to past.
Response: done

l. 166 – perhaps use “puma” instead of “lion” to maintain consistency.
Response: done

l. 172-174 – Awkward sentence, I suggest rewording.
Response: done

l. 192 – not if space use shifts as well (see major concern 1)
Response: The “all else being equal” is meant to cover the contingency. Regardless, later in the Discussion I added “but reserves in our study area are not large, so opportunity for spatial shifts is limited.” Again, I wish to make clear that we make no claims that spatial shifts are impossible. It is just that we feel our interpretation of the data is defensible given what we know about the systems and the species in it. I added a sentence along these lines to the very end.

We hope that you find this revision to be suitable for publication, and we thank you for your consideration.

Sincerely,

Michael A. Patten

Michael A. Patten
University of Oklahoma
–Presidential Professor, Oklahoma Biological Survey
–Adjunct Professor, Department of Biology
–Graduate Liaison, Environmental Studies
